# Prognostic Value of Texture Analysis Based on Pretreatment DWI-Weighted MRI for Esophageal Squamous Cell Carcinoma Patients Treated With Concurrent Chemo-Radiotherapy

**DOI:** 10.3389/fonc.2019.01057

**Published:** 2019-10-17

**Authors:** Zhenjiang Li, Chun Han, Lan Wang, Jian Zhu, Yong Yin, Baosheng Li

**Affiliations:** ^1^Shandong Cancer Hospital and Institute, Shandong First Medical University and Shandong Academy of Medical Sciences, Shandong Cancer Hospital, Jinan, China; ^2^Fourth Hospital of Hebei Medical University, Shijiazhuang, China

**Keywords:** esophageal squamous cell cancer, texture analysis, magnetic resonance imaging, diffusion-weighted magnetic resonance imaging, chemo-radiotherapy

## Abstract

**Purpose:** The purpose of the research was to assess the prognostic value of three-dimensional (3D) texture features based on diffusion-weighted magnetic resonance imaging (DWI) for esophageal squamous cell carcinoma (ESCC) patients undergoing concurrent chemo-radiotherapy (CRT).

**Methods:** We prospectively enrolled 82 patients with ESCC into a cohort study. Two DWI sequences (b = 0 and b = 600 s/mm^2^) were acquired along with axial T2WI and T1WI before CRT. Two groups of features were examined: (1) clinical and demographic features (e.g., TNM stage, age and sex) and (2) changes in spatial texture characteristics of the apparent diffusion coefficient (ADC), which characterizes gray intensity changes in tumor areas, spatial pattern and distribution, and related changes caused by CRT. Reproducible feature sets without redundancy were statistically filtered and validated. The prognostic values associated with overall survival (OS) for each parameter were studied using Kaplan-Meier and Cox regression models for univariate and multivariate analyses, respectively.

**Results:** Both univariate and multivariate Cox model analyses showed that the energy of intensity histogram texture (IHIST_energy), radiation dose, mean of the contrast in distance 1 of 26 directions (m_contrast_1), extreme difference of the homogeneity in distance 2 of 26 directions (Diff_homogeneity_2), mean of the inverse variance in distance 2 of 26 directions (m_lnversevariance_2), high-intensity small zone emphasis (HISE), and low-intensity large zone emphasis (LILE) were significantly associated with survival. The results showed that 6 texture parameters extracted from the ADC images before treatment could distinguish among high-, medium-, and low-risk groups (log-rank χ^2^ = 9.7; *P* = 0.00773). The biased C-index value was 0.715 (95% CI: 0.708 to 0.732) based on bootstrapping validation.

**Conclusions:** The ADC 3D texture feature can be used as a useful biomarker to predict the survival of ESCC patients undergoing CRT. Combining ADC 3D texture features with conventional prognostic factors can generate reliable survival prediction models.

## Introduction

Esophageal squamous cell carcinoma (ESCC) is a disease with increasing incidence, and the diagnosis still carries a poor prognosis despite advances in therapy (1). Currently, chemo-radiotherapy (CRT) is the standard treatment for locally advanced unresectable ESCC. Due to tumor heterogeneity, these patients usually do not have the same response to a specific therapy. Thus, many patients may receive therapy that provides no benefit to them. Recently, a major research focus has been on how to provide individualized therapy. Individualized therapy requires the development of biomarkers to predict treatment prognosis and outcome. Imaging biomarkers, particularly those based on functional imaging techniques that can characterize biological effects at the cellular level, offer great potential to improve individualized therapy (2).

Diffusion-weighted magnetic resonance imaging (DWI) is a powerful MR functional imaging sequence sensitive to water diffusion (3) that can detect morphological changes in tumors at the molecular or cellular level. DWI has been studied for its potential to evaluate the treatment response to CRT for several types of cancers, including rectal cancer (4, 5). The quantitative apparent diffusion coefficient (ADC) map is obtained from two different b values to remove the T2 “shine-through” effects. This can allow quantitative assessment for a treatment response. The ADC can also be used to characterize hypercellularity, distinguish cystic lesion and solid regions, and monitor the change in cellularity within the tumor over time (6). A recent study found that the ADC map can be used to qualitatively assess the tumor area and detect metastatic lymph nodes (LNs) in esophageal cancer (EC) (7).

Recently, the application of texture analysis (TA) in tumor diagnostics has caught the attention of clinical researchers. Texture is an important feature of images that has been used in qualitative and quantitative classification and analysis of materials in industry and medicine. Medical applications of TA provide a quantitative means to analyze and characterize the properties of tumor tissues and their physiological and pathological stages (8). Previous studies have reported that texture analysis can predict the treatment response and predict patient survival (9–11). It was reported that 3D texture analysis can be more useful than 2D analysis in characterizing intra-tumor heterogeneity (12). In the study of ESCC, because the esophagus is a tubular organ, 3D TA is expected to provide richer spatial heterogeneity information than slice samples.

The objective of this study was to prospectively investigate the prognostic value of 3D DWI features in ESCC patients treated with CRT. By studying different types of global and regional 3D features, we evaluate their potential prognostic value in correlation with patient survival.

## Materials and Methods

### Clinical Characteristics of the Patients

Eighty-two patients with newly diagnosed ESCC treated with CRT between 2010 and 2014 were initially enrolled in this prospective study. The inclusion criteria for the study included the following: (1) a confirmed diagnosis of ESCC with tissue pathology; (2) TNM staging according to AJCC 6th Edition, 2002; (3) a Karnofsky performance status (KPS) score >70; (4) no distant metastases under routine medical care; (5) informed consent to have DWI examinations before and during the course of CRT. Ten patients were excluded from the study because of a contraindication to MRI examination, such as those with pacemakers, metal objects, or a claustrophobic disorder. The clinical and treatment characteristics of the qualified 72 patients are summarized in Table 1. The mean age at the time of diagnosis was 62.8 ± 9.1 years (median, 62.5 years; range, 45–84 years). The male patients comprised 69.4%. Fifty-nine patients had T3 or T4 primary lesions, 53 patients were determined to have N1 (61%) with lymph node metastases, and 11 and 8 patients were at the N0 and N2 stages, respectively. No patient had distant metastases (Table 1).

**Table 1:** Clinical and treatment characteristic.

**Prognostic factors**	***N***	**%**
No. of patients	72	
**AGE**		
> = 62	38	52.8
<62	34	47.2
**SEX**		
Male	50	69.4
Female	22	30.6
**LESION LENGTH**		
< = 5.5 cm		
>5.5 cm		
**PRIMARY SITE**		
Cervical	2	2.8
Upper esophagus	24	33.3
Middle esophagus	35	48.6
Lower esophagus	11	15.3
**TNM STAGE**		
T2	13	18.1
T3	20	27.8
T4	39	54.2
N0	11	15.3
N1	53	73.6
N2	8	11.1
M0	72	100
**Treatment characteristics**		
**RADIATION DOSE**		
50 Gy at 2 Gy/fx	1	1.4
54 Gy at 1.8 Gy/fx	5	6.9
54 Gy at 2 Gy/fx	2	2.8
56 Gy at 2 Gy/fx	1	1.4
57.6 Gy at 1.8 Gy/fx	1	1.4
59.4 Gy at 1.8 Gy/fx	4	5.6
60 Gy at 2 Gy/fx	47	65.3
61.2 Gy at 1.8 Gy/fx	5	6.9
63 Gy at 2 Gy/fx	6	8.3
**RADIATION TYPE**		
IMRT	58	80.6
3DCRT	14	19.4

*3DRT, 3 dimensional conformal radiation therapy; IMRT, intensity modulated radiation therapy; fx, fraction*.

### MRI Acquisition

**Figure 1:**
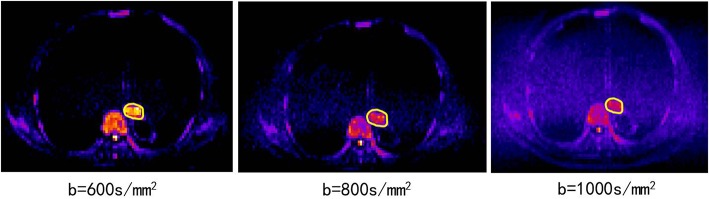
The DWI scans of b = 600, 800, 1,000 s/mm^2^ from the same patient (82 years old, male, T3N0M0).

MR imaging in expiration breath-hold was performed before starting the treatment for tumor staging. The following imaging protocols were used:

- 2D T1-weighted fast low-angle shot (FLASH) sequence (TR/TE, 140/2.5 *ms*; flip angle, 70°; slice number, 24; gap: 5 mm; matrix, 512 ×384; field of view (FOV), 380 ×285 mm);
- Axial TSE T2-weighted MRI (T2WI; slice thickness, 4 mm; slice number, 24; gap: 5 mm; TR/TE, 1580/72 *ms*; flip angle, 140°; matrix: 512 ×512; FOV, 400 ×400 mm);
- DWI obtained in the axial planes using a single-shot spin-echo echo planar (SE-EPI) technique (TR/TE, 6800/70 ms; slice thickness, 4 mm; zero gap; matrix, 128 ×88; FOV, 430 ×295 mm); the b values (diffusion-sensitive factor) of 0 and 600 s/mm^2^ were selected according to a previous pathological study at our institution (13), showing that the tumor lengths measured using a DWI scan of b = 600 s/mm^2^ were close to the real tumor lengths based on a surgical specimen with a high concordance with pathology. As shown in Figure 1, the DWI scan with b = 600 s/mm^2^ has a good image quality relative to the other DWI scans with b = 800 s/mm^2^ or 1,000 s/mm^2^.

Motion artifacts were minimized by acquiring all images with breath hold in the expiration phase. Because the tumor volumes could change slightly at the breath hold, two DWI scans with b = 0 and b = 600 were acquired in one cycle, improving the estimation of signal decay. This was expected to provide sufficient imaging information to describe tumor heterogeneity.

### Image Preprocessing

An image preprocessing procedure was performed that included tumor segmentation and intensity normalization. The MRI data set in the DICOM format was imported into MATLAB (The Math Works Inc., Natick, MA). An in-house-developed radiomics image analysis program implemented in MATLAB was used for TA (available for share, https://pan.baidu.com/s/1Tl_PsXrQj-OBJt-1cNjaZQ). The method used in this study is described in the Data Supplement. To perform reliable measurements, as suggested by Collewet et al. (14), the MRI data were kept in the raw data form, and voxels within the tumor region with intensities outside the range μ ± 3δ were excluded in subsequent texture computations. Voxel intensity values were typically resampled in four discrete values (16, 32, 64, or 128):

(1) $$\begin{array}{l} p\left(x\right)=\left[Range\times \frac{I\left(x\right)-\underset{i\in \Theta }{\min i}}{\underset{i\in \Theta }{\max i}-\underset{i\in \Theta }{\min i+1}}\right] \end{array}$$

where “Range” is the discrete values chosen (16, 32, 64, or 128), *I*(*x*)is the intensity of the original image, and Θ is the set of pixels in the delineated area. The use of different resampling schemes was tested. As discussed in the Data Supplement, 32 discrete values for renormalization produced the most reliable results.

### Tumor Delineation Using MRI Data

**Figure 2:**
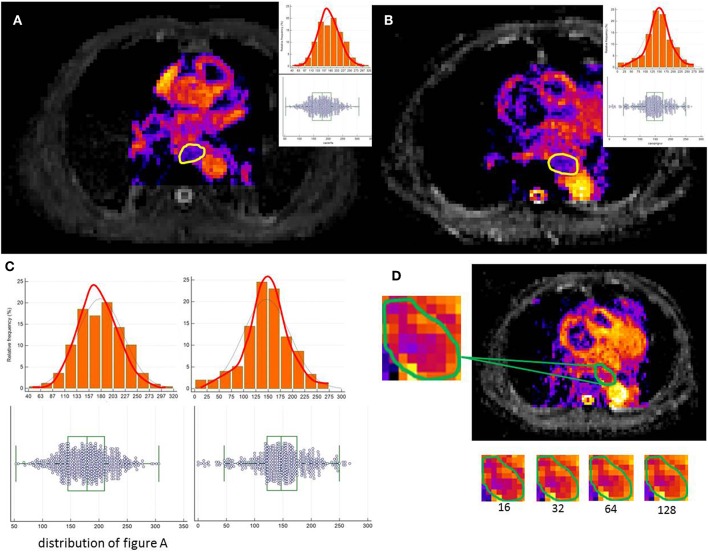
A free-hand ROI was drawn along the border of the low signal of the tumor on the b = 600 images to cover the entire tumor area of each selected slice. **(A,B)** show image heterogeneity by the histogram **(C)**. **(D)** illustrates a tumor slice of the resulting resampled ROI for each of these discretization ranges.

The tumor was delineated based on abnormal regions from T2-weighted imaging (T2WI), DWI and ADC maps. Axial ADC maps were generated using an Extended Siemens MR Workspace workstation. The lesions showed relatively higher signals on DWI maps and lower signals on ADC maps. Pre-CRT MRI was first evaluated using the combination of corresponding T2WI and ADC images and matching between DWI and ADC to correctly position the regions of interest (ROIs) in the primary tumor. Axial T2 images in the same plane were referenced by the observers due to a lack of anatomic details because of the low signal-to-noise ratio (SNR) on DWI or ADC. Therefore, the registration accuracy between ADC/DWI and T1WI/T2WI was important for the process, which relies on careful registration by the alignment of local bone structures between ADC and T2WI images. The ROI was drawn along the border of the low signal of the tumor on the b = 600 mm/s^2^ ADC images to cover the entire tumor area of each selected slice, avoiding regions with distortions or artifacts by verifying the lesion boundaries on T2WI. Delineation of the lesions was performed independently by two observers. Manual delineations were performed using MIM software (MIMvista Corp, Cleveland, OH). Independent samples *t*-test or the Kruskal-Wallis H test, where appropriate, was used to assess the differences between the features generated by reader 1 and those by reader 2, as well as between the twice-generated features by reader 1. Inter- and intra-class correlation coefficients (ICCs) were used to evaluate the intra- and inter-observer agreements of the contour agreement and feature extraction. ICC values >0.80 indicated good agreement. A free-hand ROI was drawn along the border of the low signal of the tumor on the b = 600 images to cover the entire tumor area of each selected slice, by referencing T2WI to verify the lesion boundaries and ensure inclusion of the entire tumor area (Figure 2).

### Texture Analysis

From ADC images, four subset features were extracted (Table 2) to characterize tumor heterogeneity at global and regional levels using first-order and higher order statistics. These parameters were used to predict the patient response to CRT and survival. Global information was described by intensity histogram parameters (IHIST), including variance, mean, energy, entropy, skewness, and kurtosis, while regional information was characterized by intensity size-zone variability features (ISZFs). Regional heterogeneity information included intensity variability in the size and tumor zones [see Tixier et al. (15) for the mathematical definition of the regional heterogeneity formula used in this study]. Local heterogeneity information was derived using the co-occurrence of the gray-level co-occurrence matrix (GLCM) and gray-level gradient co-occurrence (GLGCM). Twenty-six gray-level co-occurrence matrices were computed in the direction of the 26 uniform distributions on the sphere from each voxel data area. GLGCM was acquired in the original ADC image, and the corresponding gradient image of the ADC image and 15 Haralick features (16) were extracted from GLGCM. To obtain isotropy properties, the mean value and difference in the maximum and minimum value from the same Haralick features were computed in 26 directions and four distances (1, 2, 4, and 8 voxel distance). One hundred twenty-eight GLCM features were constructed by 64 mean values and 64 extreme difference values. Similarly, 60 gray gradient features were extracted from GLGCM. One hundred twenty-eight local heterogeneity features of co-occurrence matrices characterized variations in the intensity between consecutive voxels. For texture reporting with GLCM, the notation convention “method”_“feature”_“number” was used; for example, the mean of the contrast in distance 2 of 26 directions would be identified by m_contrast_2 and the extreme difference of cluster shade in distance 1 of 26 directions would be identified by Diff_clustershade_1. The GLGCM features were identified by GLGCM_“feature”_“distance”; the small gradient emphasis in distance 4 would be identified by GLGCM_Small gradient emphasis_4. The histogram-related features would be abbreviated by IHIST_“feature”; for instance, the histogram energy feature would be identified by IHIST_energy. The detailed feature information is shown in Table 2. The algorithms for texture feature extraction are described in the Data Supplement.

**Table 2:** Extracted texture features.

**IHIST**	GLCM^*^	GLGCM^*^	**ISZFs**
Mean	Mean/diff energy	Small gradient emphasis	Small zone emphasis
Variance	Mean/diff entropy	Large gradient emphasis	Large zone emphasis
Median	Mean/diff correlation	No homogeneity of gray	Gray intensity change
Maximum	Mean/diff contrast	No homogeneity of gradient	Zone size change
Minimum	Mean/diff homogeneity	Energy	Zone percentage
Up quarter value	Mean/diff variance	Mean of gray	High intensity emphasis
Down quarter value	Mean/diff mean	Mean of gradient	Low intensity small zone emphasis
Energy	Mean/diff inertia	Variance of gray	High intensity small zone emphasis
Entropy	Mean/diff cluster shade	Variance of gradient	Low intensity large zone emphasis
Skewness	Mean/diff cluster tendency	Correlation of gradient	High intensity large zone emphasis
Kurtosis	Mean/diff max probability	Entropy of gray	
	Mean/diff inverse variance	Entropy of gradient	
	Mean/diff inverse difference moment	Mix entropy	
	Mean/diff sum mean	Inertia	
	Mean/diff sum entropy	Inverse difference moment	
	Mean/diff difference entropy		

*IHIST, Intensity histogram texture; GLCM, gray level co-occurrence matrix; GLGCM, gray level gradient co-occurrence; ISZFs, Intensity size-zone variability features; diff, the extreme difference of feature*.

* *128 GLCM features are constructed by 64 mean values and 64 extreme difference values. Similarly, 60 gray gradient features were extracted from GLGCM*.

### Feature Selection Methods

In this study, 229 features in four categories were selected. Notably, not all the features required evaluation because many features would be irrelevant or redundant. Therefore, the number of features tested must be reduced by feature extraction. The three major reasons to perform feature reduction are as follows: (1) to reduce the training time; (2) to improve the robustness; and (3) to enhance the reliability.

To assess texture feature reproducibility, Fried DV's method was used to perform test-retest scans from 10 independent patients (17). The results are shown in the Data Supplement. Reproducibility of the characteristic parameters is an important characteristic in repeated experiments. In this study, a concordance correlation coefficient (CCC) value >0.9 was considered to guarantee reproducibility. Another consideration was the use of a defined “dynamic range” (DR) metric to select highly differentiated features. Similar to CCC, DR ≥ 0.9 indicates that this feature has a large dynamic range (18). The *R*^2^ of simple regression was equal to the square of the Pearson correlation coefficient. Values close to 1 indicate that the data points were close to the fitted line. These features were grouped by *R*^2^ between them. We recursively repeated the process to cover all features. We also calculated *R*^2^ between the remaining features to quantify the dependencies. Using the above methods, 38 features were chosen in the penalized model with highly reproducibility and a dynamic range.

To avoid an inadequate sample size to train and test, the “leave one out” cross validation method was used to test the model stability. Using many features, it was difficult to predict which parameters would be useful to indicate patient treatment responses and survival. Therefore, it was necessary to reduce the number of features to improve the predictability and reliability for analysis. The least absolute shrinkage and selection operator (LASSO) method was used to select the most useful predictive features from the primary data set.

The abovementioned features and clinically relevant features were entered into a penalized multivariate Cox proportional hazards model (Adaptive Elastic Net Cox model) that simultaneously performs covariate selection in addition to model development. The Adaptive Elastic Net method for the Cox model has a grouping effect (19, 20). By minimizing the opposite number of the Cox model first, and then adding the appropriate penalty, the Elastic Net estimator for the Cox model was obtained:

(2) $$\begin{array}{l} \hat{\beta }\left(EN\right)=\arg\min\{\frac{1}{n}\sum_{i=1}^{n}\left\{-\beta^{T}X_{i}+\ln\left[\sum_{j\in R_{i}}\exp\left(\beta^{T}X_{j}\right)\right]\right\}\\ \;+{\text{λ}}_{1}{\left\|\beta \right\|}_{1}+{\text{λ}}_{2}\left\|\beta \right\|2\} \end{array}$$

### Statistical Analysis

Statistical analysis was performed using SPSS19.0 (IBM, Armonk, New York, United States) for Windows and R software (version 3.2.3; http://www.Rproject.org). The R packages (hdnom v 4.1, survival v 2.39-5, penalized v 0.9-47 and survcomp v 1.20.0) were used. OS was calculated from the date of the initial diagnosis to the date of death or time for the most recent follow-up, if the patients were still alive. The reported statistical significance levels were all two-sided, with statistical significance set at 0.05.

## Results

### Overall Therapeutic Response and Survival

After the completion of CRT, an overall therapeutic response (TE) was estimated according to the RECIST 1.1 standard (21). Thirty-six patients (50%) were determined to have a complete response (CR), and 36 (50%) patients had a partial response (PR). The overall effective response rate was 100.0%.

All patients were followed up for over 1 year, and 27 patients (37.5%) were followed over 2 years. The median follow-up time was 16.5 months. The 1 and 2 year OS rates for all patients were estimated at 72.2 and 34.7%, respectively. According to the overall treatment response (CR, PR), the 1 and 2 year survival rates of CR patients were 86.1 and 38.9%, respectively, and those of PR patients were 58.3 and 30.1%, respectively. Significant differences were found between the two groups (log-rank test; χ^2^ = 4.153, *P* = 0.042).

### Prognostic Value of ADC Radiomics Data

The possible association of ADC map features with survival was explored by Kaplan-Meier survival analysis. No significant correlation was found between any ADC value measurement (ADC_mean_, ADC_up_, ADC_down_, ADC_min_, ADC_max_) in ESCC patients undergoing CRT (*P* = 0.224, 0.534, 0.549, 0.328, 0.369). The results of the log-rank analysis of conventional prognostic factors for OS in univariate analysis are given in Table 3.

**Table 3:** Conventional prognostic factors for patients.

**Variables**	**OS,%**		***P*-value**	**Hazard ratio**	**95% CI**	
	**1 year**	**2 year**			**Lower**	**Upper**
**GENDER**						
Male	74	34		1		
Female	68.2	36.4	0.325	0.710	0.360	1.403
**AGE**						
> = 62	66.7	25		1		
<62	77.8	44.4	0.168	1.002	0.991	1.055
**TUMOR SITE**						
Cervival	50	50		1		
Upper esophagus	79.2	33.3	0.884	0.851	0.098	7.356
Middle esophagus	71.4	34.3	0.626	1.284	0.469	3.513
Lower esophagus	63.6	36.4	0.499	1.394	0.531	3.658
**PATHOLOGY LESION LENGTH**						
< = 5 (40)	77.5	35		1		
>5 (32)	65.6	34.4	**0.005**	1.149	1.042	1.268
**T STAGE**						
T2	0.85	0.38		1		
T3	0.75	0.3	0.9042	1.0763	0.4629	2.5024
T4	0.66	0.36	0.2153	1.6345	0.7702	3.4688
**N STAGE**						
N0	70	40		1		
N1	73.6	34.0	0.835	1.135	0.345	3.728
N2	77.8	44.4	0.741	1.172	0.458	2.996
TNM stage I–II	78.1	34.4		1		
III	67.5	35	0.120	1.274	0.939	1.727
**GTV**						
< = 40.35	83.3	36.1		1		
>40.35	61.1	33.3	**0.015**	0.476	0.261	0.868
**TE**						
CR	86.1	38.9		1		
PR	58.3	30.6	**0.042**	1.851	1.024	3.346
**RADIATION DOSE**						
> = 60	81.5	40.7		1		
<60	44.4	16.7	**0.050**	0.916	0.807	1.015
**RADIATION TYPE**						
IMRT	69.0	32.8		1		
3DCRT	85.7	42.9	0.906	1.043	0.516	2.108

*3DRT, 3 dimensional conformal radiation therapy; IMRT, intensity modulated radiation therapy; GTV, Gross Tumor Volume; TE, therapeutic effect. The bold values show that the P ≤ 0.05*.

Age, sex, tumor site, TNM stage, and treatment type were not significant prognostic factors according to the results of univariate analysis. In univariate analysis, the GTV (Gross Tumor Volume size), pathology lesion length, therapeutic effect and radiation dose were significant prognostic factors. Univariate analysis of image texture showed that the IHIST_energy, m_contrast_1, m_Cluster shade_2, Diff_Clusetr Tendency_2, Diff_homogeneity_2, m_lnversevariance_2, Small gradient emphasis_1, GLGCM_small gradient emphasis, high-intensity small zone emphasis (HISE) and low-intensity large zone emphasis (LILE) demonstrated a statistically significant difference in association with the OS rates.

### Feature Selection

Thirty-eight texture features were reduced to 6 nonzero coefficients in the LASSO model with potential predictors based on 72 patients in the primary cohort. The detailed results used in this study were reported in the Data Supplement.

To further define the predictive values of ADC, multivariate Cox regression model analysis was performed using adjusted clinical factors. Table 4 lists the multivariate analysis results.

**Table 4:** Multivariate analysis of prognostic factor for patients with ESCC.

**Variables**	**B**	**SE**	**Wald**	**df**	***P*-value**	**Hazard ratio**	**95% CI**	
							**Lower**	**Upper**
Radiation dose	−0.125	0.067	5.112	1	0.026	1.211	0.925	1.326
IHIST_energy	−0.056	0.021	7.482	1	0.007	0.952	0.911	0.995
m_contrast_1	0.146	0.028	19.47	1	0.001	1.152	1.128	1.195
Diff_homogeneity_2	−0.022	0.002	8.824	1	0.003	0.963	0.941	0.981
m_Inverencevariance_2	0.036	0.014	4.06	1	0.034	1.042	1.002	1.13
HISE	−0.053	0.018	8.016	1	0.004	0.942	0.913	0.952
LILE	0.067	0.033	9.735	1	0.003	1.085	1.033	1.139

*IHIST_energy, the energy of intensity histogram texture; m_contrast_1, the mean of contrast in distance 1 of 26 directions; Diff_homogeneity_2, the extreme difference of homogeneity in distance 2 of 26 directions; m_Inverencevariance_2, the mean of inverse variance in distance 2 of 26 directions; HISE, high intensity small zone emphasis; LILE, low intensity large zone emphasis*.

### Validation of Model Performance

**Figure 3:**
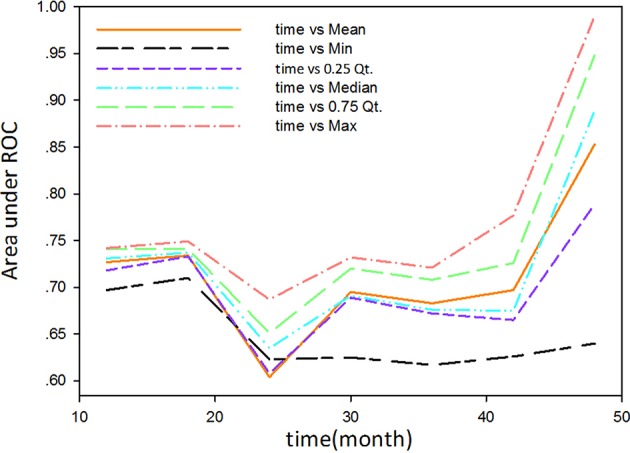
The mean, median, maximum, minimum, and 25 and 75% quartiles of time-dependent AUC at each time point across all fold predictions.

**Figure 4:**
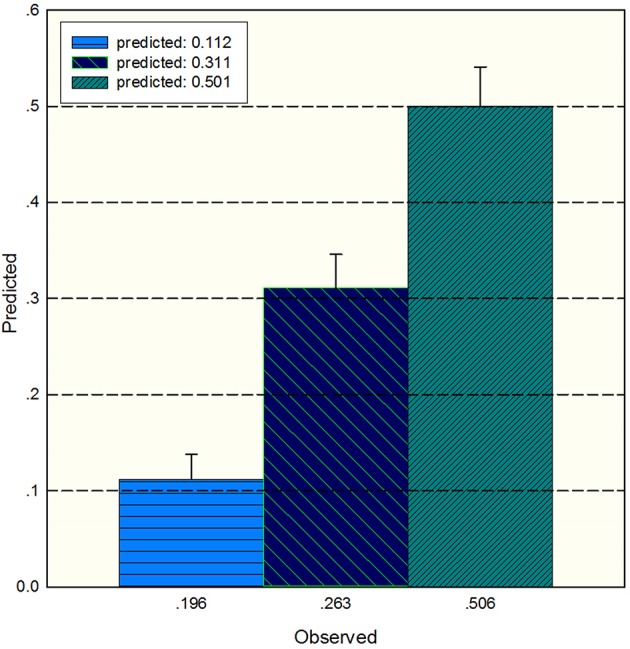
The median of the predicted survival probability and the median of the observed survival probability by the Kaplan-Meier method with 95% CI. The x axis depicts the observed value; the y axis depicts the predicted values in the corresponding point.

**Figure 5:**
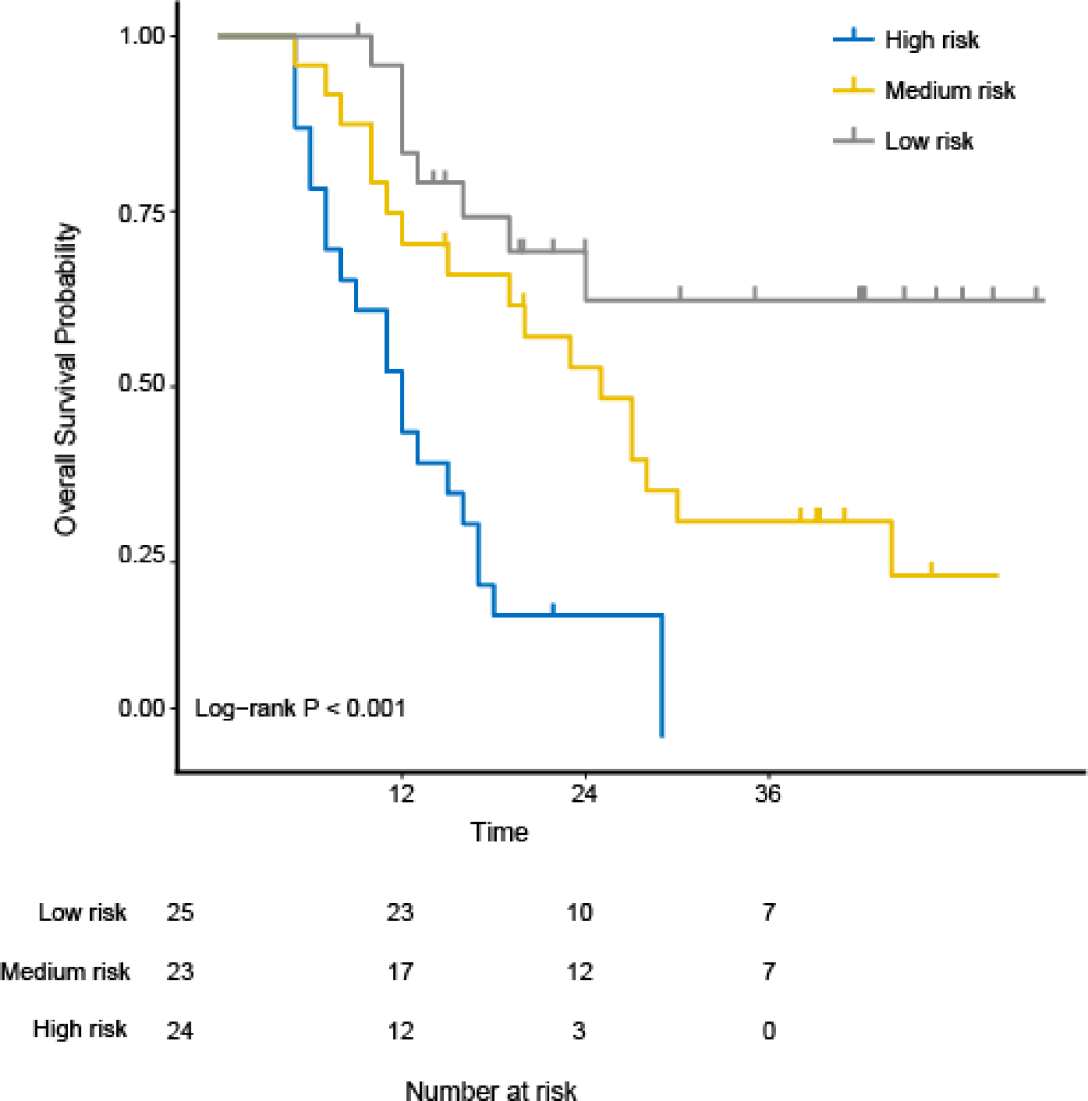
The Kaplan-Meier survival curves and evaluation of the number at risk from 1 to 3 years for the three risk groups using the “hdnom” package in R software. The *p*-value of the log-rank test is 0.00773.

The study used the “hdnom” package to assess the model performance by time-dependent AUC using the “leave one out” cross-validation method. We validated the Adaptive Elastic Net multivariate Cox model performance every 6 months. Figure 3 shows the mean, median, maximum, minimum, and 25 and 75% quartiles of time-dependent AUC at each time point across all fold predictions. The median and mean values could be considered the bias-corrected estimation of the model performance. The “leave one out” validation could ensure robust results. The figure shows that the median and mean values at each evaluation time point were relatively close. The results showed that the model had a relative high AUC value at each time point. The study used resampling methods of “leave one out” cross validation for internal model calibration. We split the samples into three risk groups according to the adaptive Elastic Net multivariate Cox model. The model calibration results (median of the predicted survival probability; median of the observed survival probability by the Kaplan-Meier method with 95% CI) are shown in Figure 4. The C-index for the prediction model was 0.720 (95% CI: 0.713 to 0.731) for the primary cohort, which was confirmed to be 0.715 (95% CI: 0.708 to 0.732) via bootstrapping validation. We used the Kaplan-Meier survival curve and values in the risk table to further analyze the survival differences among different risk groups. Here, we plotted the Kaplan-Meier survival curve and assessed the amount of risk in three risk groups from 1 to 3 years (Figure 5; χ^2^ = 9.7, Log-rank *P* = 0.00773).

## Discussion

DWI is a powerful MR sequence that provides unique information related to tumor cellularity and the integrity of the cellular membrane. The technique can be applied widely to detect and characterize tumors and to monitor the treatment response (6). The ADC map can be acquired by two DWIs (e.g., b values of 0 and 600 mm/s^2^) using an MR workstation. The ADC map is independent of the magnetic field strength and can overcome the effects of T2 shine-through, thus allowing more meaningful comparison of the results. We also performed experiments using 800 mm/s^2^ and 1,000 mm/s^2^ (Figure 1). However, the results were not reliable, the stability of the parameters was not high, and the repeatability was not good. The possible reasons for the above situation may be that a higher b value will introduce much more noise in chest tumors.

Recent investigations have demonstrated that the pretreatment ADC value may be applied as a biomarker to predict and detect early the treatment response in ESCC, but the results remain controversial. Koyama et al. (22) reported that tumors with a lower pretreatment ADC value and a higher signal intensity at DWI responded better to treatment. Koh et al. (6) discussed the mechanism of this phenomenon and showed that tumors with a high pretreatment ADC value were likely to be more necrotic than those with a low ADC value. Necrotic tumor tissues are frequently hypoxic, acidotic and poorly perfused, leading to diminished sensitivity to CRT. However, not all studies support this hypothesis. Aoyagi et al. studied 80 patients with advanced EC and found that tumors with a higher pretreatment ADC value responded better to treatment (23). They also performed a further study and found that the pretreatment ADC value was not significantly different between the responder and non-responder groups (24). Wang et al. also found no direct correlation between the pretreatment ADC value and treatment response in EC (25). The reasons for the controversy could be that simple ADC values only show limited information (one dimensional information) that only reflect variability (high or low), not including geometric distribution. Texture features can overcome the above defects, having the potential to show and quantify pixels or the voxel geometric distribution. With the development of imaging analysis, much evidence has suggested that TA can aid clinicians in cancer diagnosis (26), staging (27), prognoses (28), and response assessments (15). In our study of 82 patients with the diagnosis of ESCC, interestingly, we showed that the pretreatment DWI texture features can provide useful prognostic information for ESCC patients. Finally, previous studies were mostly focused on limited tumor areas, such as contouring ROIs in the largest section, rather than the global tumor volume. In our study, to compensate for the 2D texture feature defects (12, 29, 30), 3D texture parameters were chosen to evaluate the prediction potentials.

We first analyzed the intensity histogram features with highly reflected distribution of ADC values. The other texture features mainly focused on the local and regional scales, which were used to analyze the interrelationship between pairs of voxels and arrangement of voxels. From microscopy, the order of voxels reflected local non-uniformities. Our analysis showed that IHIST_energy, m_contrast_1, Diff_homogeneity_2, m_Inversevariance_2, HISE, and LILE have strong and independent associations with the OS rates. The IHIST_energy measures the homogeneity of gray distribution. The higher value depicts more homogeneity than the lower one. m_Contrast_1 is a measure of the contrast or amount of local variation present in the ADC. The tumor usually has a large amount of local variations present in the image compared with the normal part. The other parameters (Diff_homogeneity_2, m_Inversevariance_2) were a measure of homogeneity of the image. This represents the change in the tumor gray level and reflects the aggregation of tumor cells on the macro level. The HISE measures the joint distribution of small zones and high gray-level values. The LILE has opposite characteristic to HISE and measures the joint distribution of large zones and high gray-level values. These features represent spatial ADC variability in esophageal tumors, explaining why these ADC texture features are better prognostic factors than simple global ADC values.

The OS variation in ESCC patients treated with CRT is highly related to tumor heterogeneity due to its intra-tumor spatial variation in the cellularity, angiogenesis, extravascular, extracellular matrix, and areas of necrosis. The high tumor heterogeneity was shown to have a poorer prognosis and treatment resistance (31). Ganeshan et al. (32) found that tumor heterogeneity in EC could be reflected by TA. Our study showed that six 3D texture parameters extracted from ADC maps can distinguish among the high-, median-, and low-risk group (Log-rank χ^2^ = 9.7; *P* = 0.00773). The idea behind this performance is that texture parameters can reflect the movement of water molecules and tumor heterogeneity. This may become a major mechanism to explain why texture parameters can accurately associate with the OS of ESCC.

To ensure the model's stability, the test-retest method was used to test the selected feature stability in the feature selection step. In the model validation step, the “leave one out” cross validation for both model validation and model calibration was used. Compared with the general sampling test (splitting their data into test and validation sets), the LASSO regularization scheme was used to prevent over fitting (33).

This study has an important clinical significance. Uncertainties remain in the treatment of ESCC, including the scope of the radiotherapy target area, the dose of radiation therapy, the consolidation chemotherapy maintenance period, the assessment of the clinical effect and so on. The cause of the above uncertainty remains a lack of effective means to describe ESCC heterogeneity. The texture features combined with conventional prognostic factors may present a more accurate predictive tool. Our research showed that texture features can be used to evaluate the prognosis of ESCC after CRT at the early phase. However, our study is limited by several factors, the most important of which is the prospective nature of the assessment using a relatively small group of patients. It is necessary to expand the sample size for further study to clearly explore the relationship between the global ADC value and OS. Another limitation of this study is that the tumor regions of interest were drawn manually; inter- and intra-observer variation could be reduced if automated methods were used in the future, particularly for multicenter studies.

## Conclusions

Based on the ADC images, the texture parameters extracted by computer semi-automatic extraction are related to ESCC patient survival. This study confirms that the combination of ADC textures (histogram feature, GLCM feature, and ISZF feature) and conventional prognostic factors (radiation dose) can be used to generate robust models to predict OS. Future work needs to further verify the practical value of related parameters in clinical application.

## Data Availability Statement

The datasets for this manuscript are not publicly available because involving patient confidential information. Requests to access the datasets should be directed to zhenjli@seu.edu.cn.

## Ethics Statement

Registration number and name of registry: 20091124-2. Project: Prognostic Value of Pretreatment Diffusion Weighted Magnetic Resonance Imaging based Texture in Concurrent Chemo-radiotherapy of Esophageal Squamous Cell Cancer. Institute: Shandong Cancer Hospital Affiliated to Shandong University, Department of Radiation Oncology, the Fourth Hospital of Hebei Medical University. Research Summary: In this retrospective cohort study, we aims to include a total 100 patients with esophageal squamous cell carcinoma in Chinese population. MR imaging examination was performed before starting the treatment for tumor staging. The imaging protocol included: (1) 2D Fast Low-Angle Shot imaging (FLASH) /T1-weighted and DWI was obtained in axial planes. Privacy Highlights: patients privacy. Comments of Institutional Review Board: After carefully examination of the relative information, including researcher qualifications, research programme, CRF, and informed consents, the study was approved by the Institutional Review Board.

## Author Contributions

All authors listed have made a substantial, direct and intellectual contribution to the work, and approved it for publication.

## Conflict of Interest

The authors declare that the research was conducted in the absence of any commercial or financial relationships that could be construed as a potential conflict of interest.
