# Supplementary material for: Prognostic Value of Texture Analysis Based on Pretreatment DWI-Weighted MRI for Esophageal Squamous Cell Carcinoma Patients Treated With Concurrent Chemo-Radiotherapy
Source: Front Oncol. 2019 Oct 17;9:1057. doi: 10.3389/fonc.2019.01057 (PMC6811607; doi:10.3389/fonc.2019.01057)
Supplement: Supplementary file 1 [file Data_Sheet_1.docx]

### Appendix

**Appendix 1. Image pre-processing**


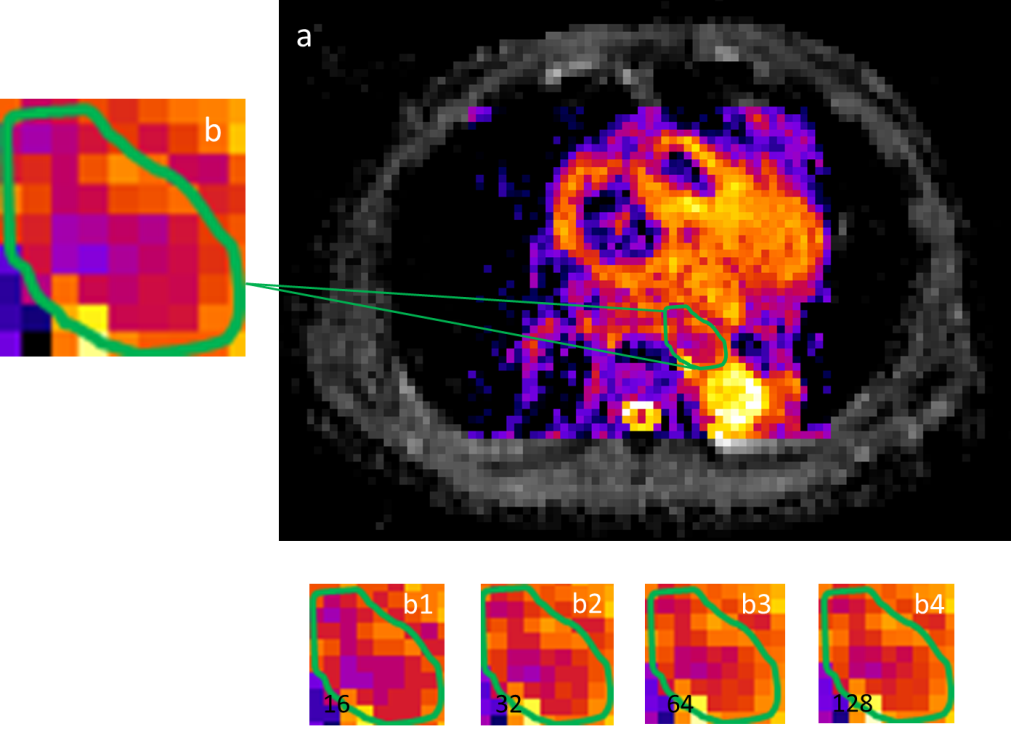


Figure 1. The figure illustrates on a tumor slice the resulting resampled ROI for each of these discretization ranges. The figure (a) shows an axial slice for a patient with ESCC; figure (b) shows the outlined area. The figure b1-b4 shows the down sampling to ranges of 16-128.


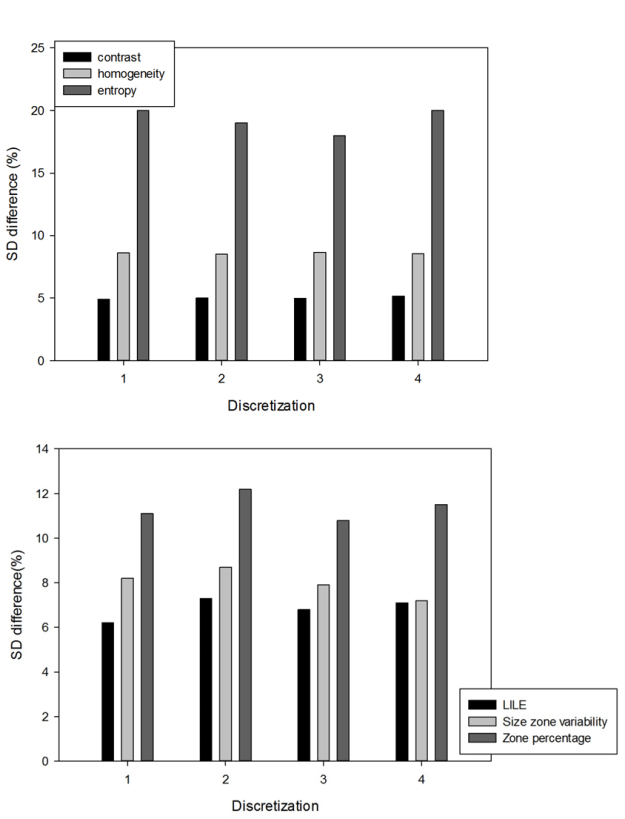


Figure 2. Plots shows the SD of mean percentage difference as characterization of discretization value for parameters derived from co-occurrence matrices (entropy, homogeneity, contrast ) (figure A showing) and intensity-size-zone matrix (figure B showing). LILZ = low-intensity large-zone emphasis. The results showed that almost all textural parameters describing local heterogeneity were insensitive to the chosen discretization values. The entropy was characterized by a mean difference of 19%±5% and 20%±8% using 16 and 128 values, respectively. On the other hand, the intensity and size variability of uniform tumor areas were largely independent (SD differences < 20%) of the discretization values, with no statistically significant differences.

**Appendix 2. Texture analysis**

Texture analysis was applied to this study using in-house texture analysis software with algorithms implemented in Matlab 2014a (Mathworks, Natick, USA). The codes are available at this website <https://pan.baidu.com/s/1sl3GqDJ> .


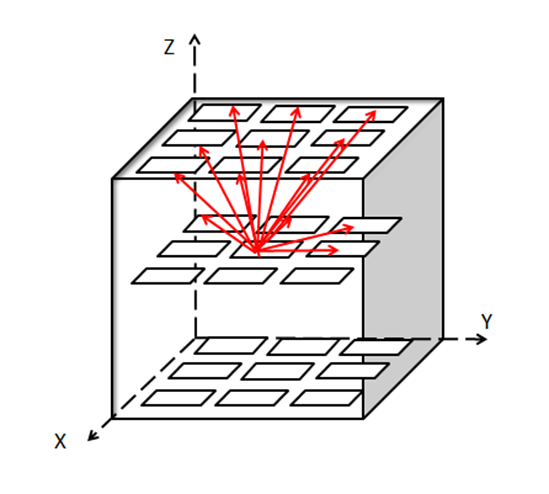


Figure 3 shows 26 uniform distributions on the spherical from each voxel data area.

A series of gray-level gradient co-occurrence matrix (GLGCM), regional heterogeneity features of intensity-size-zone matrix (ISZFs) texture features, as listed in Table S1 and Table S2. Local heterogeneity information was derived using co-occurrence of the gray level co-occurrence matrix GLCM and gray level gradient co-occurrence GLGCM.

Table S1. A summary of textural features extractable from Gray-level gradient co-occurrence matrix (GLGCM)

| Feature | Formula |
| --- | --- |
| Small gradient Emphasis | $\left( \sum_{i=1}^{N} \sum_{j=1}^{N} \frac{H\left( i,j \right)}{j^{2}} \right)/\left( \sum_{i=1}^{N} \sum_{j=1}^{N} H(i,j) \right)$ |
| Large gradient Emphasis | $\left( \sum_{i=1}^{N} \sum_{j=1}^{N} i^{2}H(i,j) \right)/\left( \sum_{i=1}^{N} \sum_{j=1}^{N} H(i,j) \right)$ |
| Gray inhomogeneous | $\left( \sum_{i=1}^{N} \left[ \sum_{j=1}^{N} H(i,j) \right]^{2} \right)/\left( \sum_{i=1}^{N} \sum_{j=1}^{N} H(i,j) \right)$ |
| Gradient gray inhomogeneous | $\left( \sum_{j=1}^{N} \left[ \sum_{i=1}^{N} H(i,j) \right]^{2} \right)/\left( \sum_{i=1}^{N} \sum_{j=1}^{N} H(i,j) \right)$ |
| Gradient energy | $\sum_{i=1}^{N} \sum_{j=1}^{N} \left[ H(i,j) \right]^{2}$ |
| Mean Gray | $\sum_{i=1}^{N} i\left[ \sum_{j=1}^{N} H(i,j) \right]$ |
| Mean Gradient | $\sum_{j=1}^{N} j\left[ \sum_{i=1}^{N} H(i,j) \right]$ |
| Gray variance | $\left\{ \sum_{i=1}^{N} \left( i-\mu_{1} \right)^{2}\left[ \sum_{j=1}^{N} H(i,j) \right] \right\}^{1/2}$ |
| Gradient variance | $\left\{ \sum_{j=1}^{N} \left( j-\mu_{2} \right)^{2}\left[ \sum_{i=1}^{N} H(i,j) \right] \right\}^{1/2}$ |
| Gradient correlation | $\frac{1}{\sigma_{1}\sigma_{2}}\sum_{i=1}^{N} \sum_{j=1}^{N} \left( i-\mu_{1} \right)\left( j-\mu_{2} \right)H(i,j)$ |
| Gray entropy | $-\left\{ \sum_{i=1}^{N} \left[ \sum_{j=1}^{N} H(i,j) \right]\cdot log\left[ \sum_{j=1}^{N} H(i,j) \right] \right\}$ |
| Gradient entropy | $-\left\{ \sum_{j=1}^{N} \left[ \sum_{i=1}^{N} H(i,j) \right]\cdot log\left[ \sum_{i=1}^{N} H(i,j) \right] \right\}$ |
| Mixture entropy | $-\sum_{i=1}^{N} \sum_{j=1}^{N} H(i,j)\cdot logH(i,j)$ |
| Gradient difference moment | $\sum_{i=1}^{N} \sum_{j=1}^{N} \left( i-j \right)^{2}H(i,j)$ |
| Gradient inverse difference moment | $\sum_{i=1}^{N} \sum_{j=1}^{N} 1/{1+\left( i-j \right)^{2}}H(i,j)$ |

Table S2. Formulas for regional heterogeneity features of intensity-size-zone matrix (ISZFs)

| Feature | Formula |
| --- | --- |
| Small zone Emphasis | $\frac{1}{∁}\left( \sum_{i=1}^{M} \sum_{j=1}^{N} \frac{Z\left( i,j \right)}{j^{2}} \right)$ |
| Large zone Emphasis | $\frac{1}{∁}\left( \sum_{i=1}^{M} \sum_{j=1}^{N} j^{2}\cdot Z(i,j) \right)$ |
| Intensity variability | $\frac{1}{∁}\left. \sum_{i=1}^{M} \left[ \sum_{j=1}^{N} \frac{Z\left( i,j \right)}{i^{2}} \right]^{2} \right.$ |
| Size zone variability | $\frac{1}{∁}\left. \sum_{j=1}^{M} \left[ \sum_{i=1}^{N} \frac{Z\left( i,j \right)}{j^{2}} \right]^{2} \right.$ |
| Zone percentage | $∁/{\sum_{i=1}^{M} \sum_{j=1}^{N} j^{2}\cdot Z(i,j)}$ |
| Low intensity emphasis | $\frac{1}{∁}\left. \sum_{i=1}^{M} \left. \sum_{j=1}^{N} \frac{Z(i,j)}{i^{2}} \right. \right.$ |
| High intensity emphasis | $\frac{1}{∁}\left. \sum_{i=1}^{M} \left. \sum_{j=1}^{N} i^{2}\cdot\right. \right.Z(i,j)$ |
| Low-intensity small-zone emphasis | $\frac{1}{∁}\left. \sum_{i=1}^{M} \left. \sum_{j=1}^{N} \frac{Z(i,j)}{i^{2}\cdot j^{2}} \right. \right.$ |
| High-intensity small-zone emphasis | $\frac{1}{∁}\left. \sum_{i=1}^{M} \left. \sum_{j=1}^{N} i^{2}\cdot j^{2} \right. \right.\cdot Z(i,j)$ |
| Low-intensity large-zone emphasis | $\frac{1}{∁}\left. \sum_{i=1}^{M} \left. \sum_{j=1}^{N} \frac{j^{2}Z(i,j)}{i^{2}} \right. \right.$ |
| High-intensity large-zone emphasis | $\frac{1}{∁}\left. \sum_{i=1∁}^{M} \left. \sum_{j=1}^{N} \frac{i^{2}Z(i,j)}{j^{2}} \right. \right.$ |
| $∁$ = number of homogeneous areas within tumor; Z = intensity size–zone matrix; M= discretization value; N = size of largest homogeneous area within tumor; z(i, j) = number of areas with intensity I and size j. | |
|  | |

**Appendix 3. The feature selection**


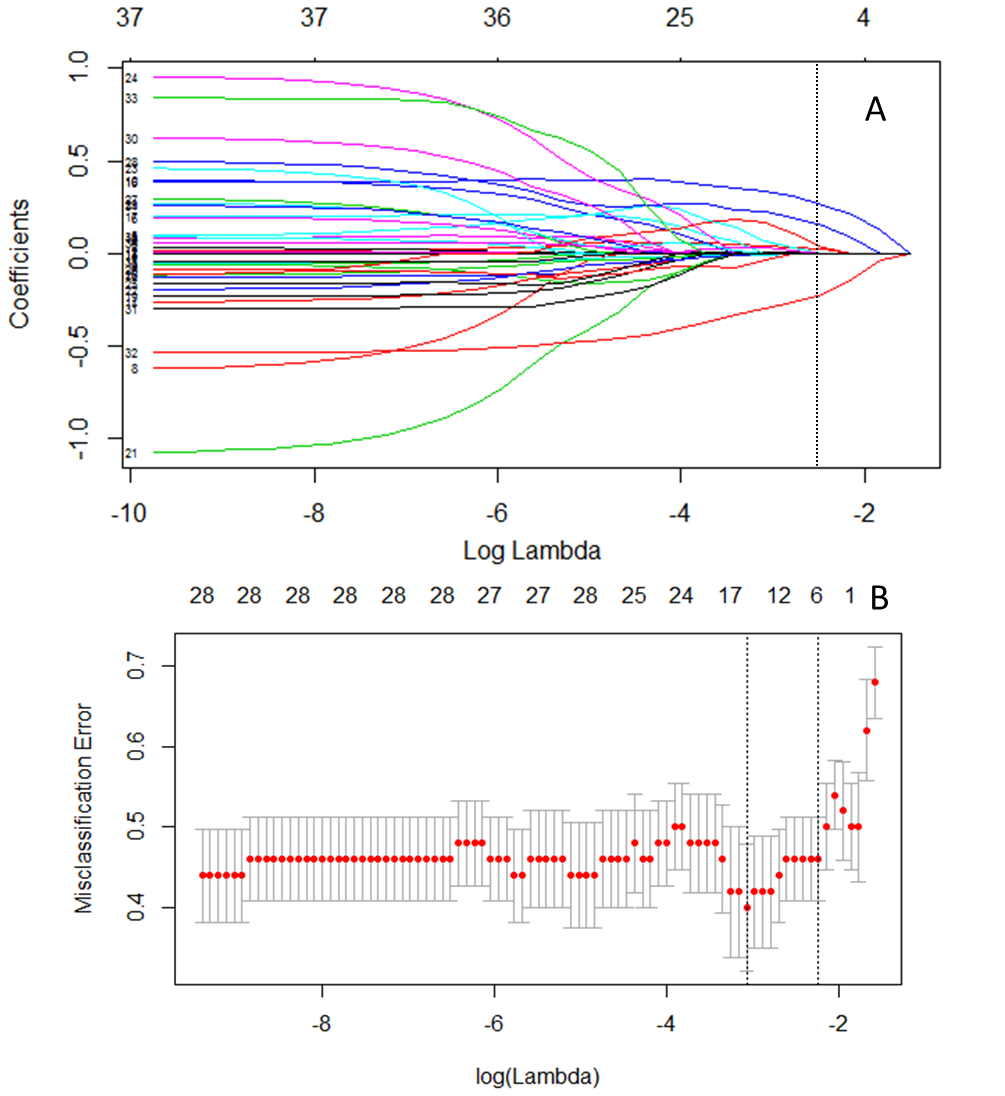


Figure 4

Texture feature selection using the LASSO binary logistic regression model. (A) LASSO coefficient profiles of the 38 texture features. A coefficient profile plot was produced against the log (λ) sequence. Vertical line was drawn at the value selected using leave one out cross-validation, where optimalλresulted in 6 nonzero coefficients. (B) Tuning parameter (λ) selection in the LASSO model used leave one out cross-validation via minimum criteria. The shrinkage of coefficient was plotted versus log(λ). Dotted vertical lines were drawn at the optimal values by using the minimum criteria and. A λvalue of 0.108 with log (λ), -2.23 was chosen according to leave one out cross-validation.
